# SPIRITUAL AND RELIGIOUS CHANGE IN LATER LIFE

**DOI:** 10.1093/geroni/igz038.1304

**Published:** 2019-11-08

**Authors:** Vern Bengtson, Merril Silverstein, Samantha Copping, Camille Endacott

**Affiliations:** 1 University of Southern California, Los Angeles, California, United States; 2 Syracuse University, Syracuse, New York, United States; 3 University of California-Santa Barbara, Santa Barbara, California, United States

## Abstract

How does religious and or spiritual engagement change with aging? Is there a “return to religion” among aging baby boomers these days? Some theories of aging posit an increase in spirituality toward the end of life, such as the psycho-developmental theories of James, Jung, Erickson, and Tornstam, while others emphasize continuity (no change). We explore this issue in a mixed-methods longitudinal study of older adults’ spiritual and religious trajectories. Data are from surveys with 981 baby boomer participants of the 45-year Longitudinal Study of Generations panel, and intensive interviews with 70 individuals 65-95 from a variety of religious and non-religious backgrounds. Results indicate complex trajectories of increase, decrease, and continuity as individuals move into their later years. We also detect a “return to religion” among baby boomers. We discuss these findings in terms of both life-course personality theories and social integration/support

